# Perinatal features of children with Silver-Russell syndrome due to 11p15 loss of methylation

**DOI:** 10.3389/fped.2024.1367433

**Published:** 2024-04-04

**Authors:** Diane Darneau, Eloïse Giabicani, Irène Netchine, Aurélie Pham

**Affiliations:** ^1^ Sorbonne Université, INSERM, Centre de Recherche Saint-Antoine, APHP, Hôpital Armand Trousseau, Endocrinologie Moléculaire et Pathologies d’Empreinte, Paris, France; ^2^ Sorbonne Université, INSERM, Centre de Recherche Saint-Antoine, APHP, Hôpital Armand Trousseau, Service de Néonatologie, Paris, France

**Keywords:** foetal growth restriction (FGR), small for gestation age (SGA), Silver–Russel syndrome, neonatal care, placental insufficiency (PI)

## Abstract

**Background:**

A diagnosis of Silver–Russell syndrome (SRS), a rare imprinting disorder responsible for foetal growth restriction, is considered for patients presenting at least four criteria of the Netchine-Harbison clinical scoring system (NH-CSS). Certain items of the NH-CSS are not assessable until the age of 2 years. The objective was to determine perinatal characteristics of children with SRS to allow an early diagnosis.

**Methods:**

We retrospectively compared the perinatal characteristics of children with SRS (*n* = 17) with those of newborns small for gestational age (SGA) due to placental insufficiency (PI) (*n* = 21).

**Results:**

Children with SRS showed earlier and more severely altered foetal biometry than SGA newborns due to PI. Twenty-three percent of patients with SRS showed uterine artery Doppler anomalies. SRS children were significantly smaller at birth (birth length <-3 SDS in 77% of cases in the SRS group vs. 15% in the PI group, *p* = 0.0001).

**Conclusion:**

The diagnosis of SRS must be evoked in the neonatal period for SGA newborns with a growth delay present from the second trimester of pregnancy, a birth length <-3 SDS and a relative macrocephaly. Doppler anomalies, classically used to orient the cause of SGA towards PI, did not rule out the diagnosis of SRS.

## Introduction

Foetal growth restriction (FGR), defined as a failure of the foetus to reach its genetically determined growth potential, is one of the most common causes of perinatal mortality and morbidity (1). The definition of FGR is debated and includes children with intrauterine growth restriction (IUGR) and those born small for gestational age (SGA), defined by a birth weight (BW) and/or birth length (BL) below the 10th percentile of a given reference by neonatologists (2, 3) or a standard deviation score (SDS) ≤2 for BW and/or BL by endocrinologists (4). In industrialised countries, the prevalence of children born SGA is estimated to be 10.8% (5).

Placental vascular insufficiency, defined as the inability of the placenta to provide sufficient nutrients and oxygen to the foetus for growth and development, is the most common cause of intrauterine growth retardation and is associated with neonatal morbidity and mortality (6).

However, FGR can result from multiple causes, such as genetic, epigenetic, or hormonal regulation (3). Parental imprinting, an epigenetic mechanism that refers to the monoallelic silencing of genes according to their parental origin, is known to play an important role in foetal growth (7). Silver-Russell syndrome (SRS) is a rare imprinting disorder characterised by foetal and postnatal growth restriction and feeding difficulties requiring specific multidisciplinary care (8). A clinical diagnosis of SRS is considered if a patient presents with at least four of the six criteria of the Netchine–Harbison clinical scoring system (NH-CSS) (8, 9), which includes pre- and postnatal growth retardation, relative macrocephaly at birth, body asymmetry, protruding forehead, and early feeding difficulties (Supplementary Table SD1, Supplemental Data). Epimutation, resulting in the loss of methylation (LOM) of the *H19*/*IGF2* intergenic differentially methylated region (IG-DMR) is identified in 50% of SRS patients (8). Certain items in the NH-CSS are not assessable until the age of 2 years, such as growth retardation or feeding difficulties. Moreover, body asymmetry can be difficult to identify in the first months of life. Without these items, children with SRS may not meet the four criteria required for a clinical diagnosis of SRS and may not undergo the molecular investigations recommended in the international consensus statement on the diagnosis and management of SRS (8). An adapted threshold for molecular testing may be required for children aged under 2 years.

Children born with SRS due to LOM of the *H19*/*IGF2* IG-DMR have severe IUGR with mean BW −3.5 SDS, mean BL −4.1 SDS with relative macrocephaly at birth (mean birth HC −0.5 SDS) (10). Children born with severe SGA (<3rd percentile) due to placental insufficiency (PI) and those with SRS share many common features during the perinatal period, such as the presence of a relatively preserved head circumference (HC) at birth. However, children born SGA due to SRS have a different evolution than children born SGA due to PI and require appropriate follow-up and management. It is therefore important to be able to make the diagnosis of SRS as early as possible.

The objective of our study was to determine perinatal characteristics of children with SRS in order to differentiate children with SRS and those with severe FGR due to vascular PI, the main differential diagnosis in neonatal period, to allow earlier clinical and/or molecular diagnosis of SRS and its subsequent management.

## Materials and methods

### Study design and participants

We conducted a non-interventional study based on data collected from medical and obstetrical records and retrospectively compared the perinatal characteristics of children with SRS to those of children born SGA due to PI.

All SRS patients had molecularly confirmed SRS (*IGF2*/*H19*: IG-DMR LOM) and were either followed in our clinic or referred by other clinical centres for molecular analysis. Each patient had been examined by a geneticist and/or paediatric endocrinologist. A clinical file, including extensive clinical data, growth charts, a detailed phenotypic description, and pictures, was completed for all patients.

SGA children were considered to have SGA due to PI when they had a BW and/or BL <3rd percentile with a HC at birth >10th percentile, histologically confirmed placental vascular lesions (hypotrophic placenta with foci of thrombosis or haemorrhage or territories of placental malperfusion) and achieved catch up growth without growth hormone therapy before the age of two years. All patients were followed in our clinic.

### Clinical assessment and definition

The estimated foetal weight (EFW) was calculated according to Hadlock's formula (11). Foetal biometric parameters (abdominal circumference, femoral length, biparietal diameter) are expressed as a percentile according to the French College of Foetal Ultrasound reference curve.

Doppler ultrasound data, for assessing placental function, included umbilical artery resistance index and uterine artery resistance index and functional parameters such as Doppler waveform analysis (umbilical artery).

Length, weight, and HC at birth are expressed as a percentile according to Audipog morphometry curves (12) and as a SDS according to Usher and McLean charts (13). Post-natal growth parameters are expressed as a SDS according to Sempé French charts (14). The body mass index (BMI) is expressed as a SDS according to Rolland-Cachera French charts (15).

The NH-CSS was applied to each of the SRS patients (9). This scoring system defines a suspicion of SRS if at least four of the six following criteria are met: being born SGA (SDS ≤−2.0 for BW and/or BL for gestational age), postnatal growth failure (SDS ≤−2.0 for height at 24 months or SDS ≤−2.0 for height from the midparental target height), relative macrocephaly at birth (SDS ≥1.5 for HC at birth above the BW and/or BL), protruding forehead (forehead projecting beyond the facial plane on a side view as a toddler), body asymmetry and low BMI (SDS ≤−2.0 for BMI at 24 months), and/or feeding difficulties defined by the use of a feeding tube and/or cyproheptadine for appetite stimulation.

### Statistical analysis

Qualitative variables are described as numbers (percentage) and quantitative variables as medians (IQR). The characteristics of SRS and PI-IUGR patients were compared using Fisher's exact tests performed for qualitative variables and Mann-Whitney tests for quantitative variables. *P* values of <0.05 were considered statistically significant. All analyses were performed using GraphPad Prism 6.

### Ethical approval

This study received approval by the Research Ethics Committee of the French Pediatric Society (Opinion number: CER_SFP_2020_125_2) on February 11, 2021. Written informed consent for participation was received from parents of all patients, in accordance with French national ethics rules for patients recruited in France.

## Results

### Patient characteristics

Seventeen SRS patients with *IGF2*/*H19*: IG-DMR LOM and 21 with SGA due to PI were included. The characteristics of the patients are presented in Table 1.

**Table 1:** Patient characteristics.

	PI children (*N* = 21)	SRS children (*N* = 17)	*p*
Age at inclusion (in months)			
Mean	61	89	0.50
Min-max	38–83	11–187	
Sex			
Male	13 (62%)	9 (53%)	0.75
Female	8 (38%)	8 (47%)	
Pregnancy			
Single	14 (67%)	15 (88%)	0.15
Multiple	7 (33%)	2 (12%)	
Birth term (weeks of amenorrhea)			
Median	37	37	1
Min-max	27–41	28–39	
Birth term (weeks of amenorrhea)			
≥37 WA	13 (62%)	10 (59%)	0.74
32–36 + 6 WA	6 (28%)	4 (23%)	
28–33 + 6 WA	1 (5%)	2 (12%)	
24–27 + 6 WA	1 (5%)	1 (6%)	
Medically assisted procreation			
No	6 (29%)	1 (6%)	0.64
Yes	15 (71%)	7 (41%)	
NA	0 (0%)	9 (53%)	
Labour			
Spontaneous	3 (14%)	2 (12%)	1
Induced	18 (86%)	15 (88%)	
Delivery			
Vaginal delivery	6 (29%)	6 (35%)	0.74
Caesarean section	15 (71%)	11 (65%)	
Amniocentesis	3 (14%)	12 (71%)	0.01*

NA, data not available; WA, weeks of amenorrhea.

* *p* < 0.05.

All SRS patients had a NH-CSS ≥4/6 at the time of inclusion (Supplementary Table SD2). The median age at molecular diagnosis of SRS was 16 months (4 months to 13 years), with a mean methylation index ranging from 8% to 40%. Eighty-eight percent of the SRS children were under 4 years of age at the time of diagnosis.

Twelve SRS patients (71%) and three PI patients (14%) underwent an invasive prenatal investigation to determine the cause of SGA (FISH (fluorescence *in situ* hybridisation), karyotype, CGH (genomic hybridisation array), and CMV PCR (cytomegalovirus polymerase chain reaction).

### Foetal biometric parameters

**Figure 1:**
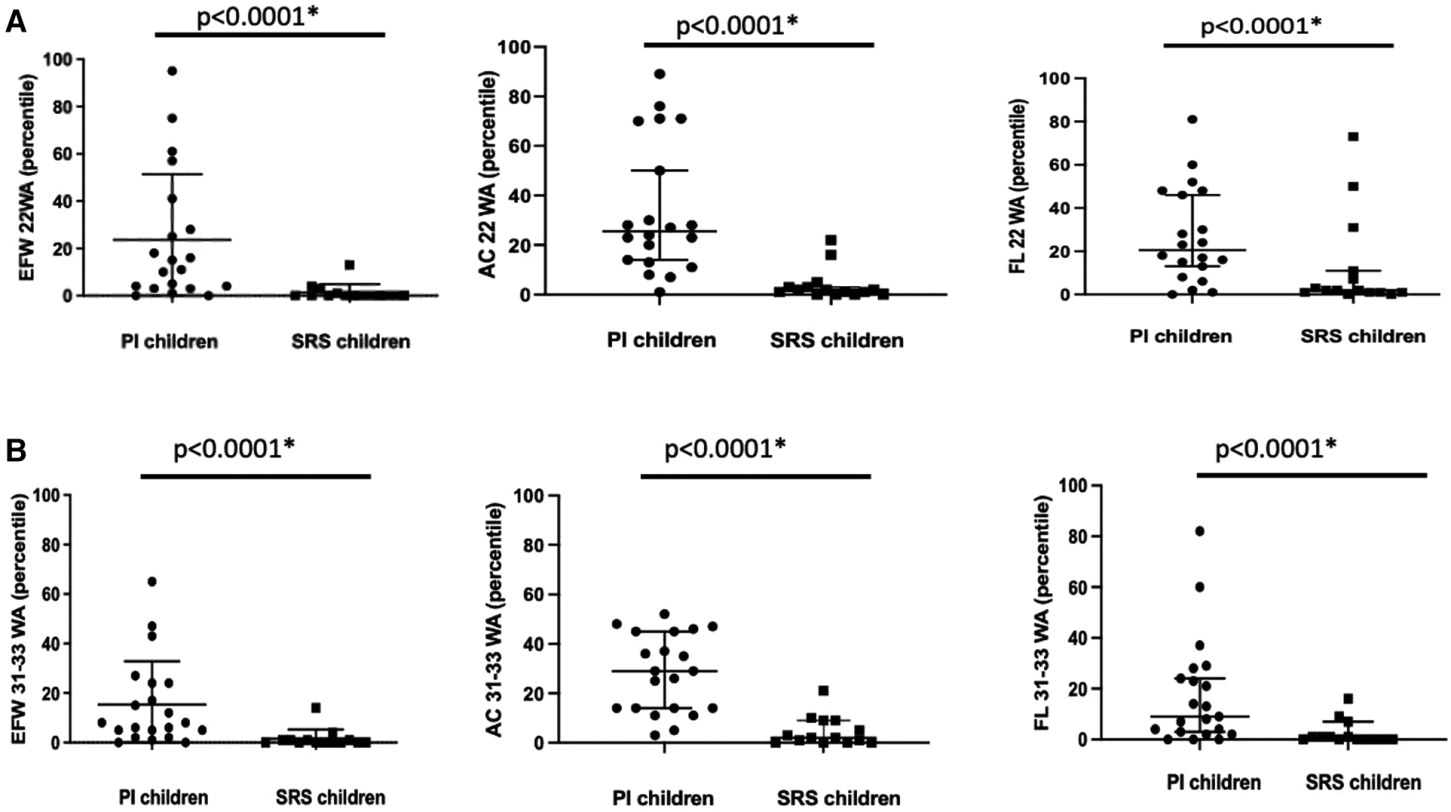
Comparison of the estimated foetal weight (EFW), abdominal circumference (AC), and femur length (FL) in grams and/or millimetres and percentiles at obstetric ultrasound performed at 22 WA (**A**) and 31-33 WA (**B**) between the two groups. Bars represent the median and 95% confidence interval. *p < 0.05.

The estimations of foetal biometric parameters are presented in Figure 1 and Supplemental Data (Supplementary Tables SD3, SD4). Patients with SRS had lower foetal biometric parameters than those with SGA due to PI. Indeed, from the obstetric ultrasound performed at 22 weeks of amenorrhea (WA), patients with SRS had a lower estimated foetal weight (EFW) than children in the PI group (365 vs. 494 g, *p* < 0.001; 0 vs. 13th percentile, *p* < 0.001), as well as a lower abdominal circumference (149 vs. 175 mm, *p* < 0.001; 2nd vs. 25th percentile, *p* < 0.001) and shorter femoral length (34 vs. 38.3 mm, *p* = 0.01; 2nd vs. 20th percentile, *p* = 0.01).

The same results were observed at the obstetric ultrasound performed between 31 and 33 WA, with a lower percentile EFW for SRS patients than that for PI children (0 vs. 8th percentile, *p* < 0.001), as well as a lower estimated abdominal circumference (2nd vs. 29th percentile, *p* < 0.001) and shorter femoral length (0.5 vs. 9th percentile, *p* = 0.01).

There was no statistically significant difference between the two groups concerning the measurement of the biparietal diameter or HC at the obstetric ultrasound performed at 22 WA or between 31 and 33 WA.

### Doppler velocimetry

There was no significant difference in Doppler velocimetry between the two groups (Table 2). Twenty-three percent of SRS patients had abnormal uterine artery Doppler velocimetry with a resistance index (RI) >0.65 and the presence of diastolic notches at obstetric ultrasound performed at 22 WA.

**Table 2:** Comparison of Doppler velocimetry between the two groups.

	PI children (*N* = 17)	SRS children (*N* = 21)	*p*
Umbilical artery resistance index at obstetric ultrasound performed between 31 and 33 WA (*N*: 0.66 ± 0.05)	0.68 [0.52; 0.95]	0.69 [0.63; 1.02]	0.28
Uterine artery resistance index >0.65 at obstetric ultrasound performed at 22 WA	7/13 (54%)	3/13 (23%)	0.23
Uterine artery resistance index >0.65 at obstetric ultrasound performed between 31 and 33 WA	2/9 (22%)	2/4 (50%)	0.53
Diastolic notch at obstetric ultrasound performed at 22 WA	3/13 (23%)	3/13 (23%)	1
Diastolic notch at obstetric ultrasound performed between 31 and 33 WA	3/9 (33%)	2/4 (50%)	0.99

WA, weeks of amenorrhea; [xx; xx]: Min—max.

Two SRS patients had abnormal uterine artery Doppler velocimetry with a RI >0.65 at obstetric ultrasound performed between 31 and 33 WA and the presence of diastolic notches.

### Placental pathology analysis

Placental pathology reports were available for two SRS patients (one patient had abnormal uterine Doppler velocimetry). No histologically confirmed placental vascular lesions were described for these two patients. One was described as hypotrophic with early chorioamniotitis without a foetal inflammatory response and the other had focal lesions of chronic lymphocytic villitis of unknown aetiology.

### Biometric parameters at birth

**Figure 2:**
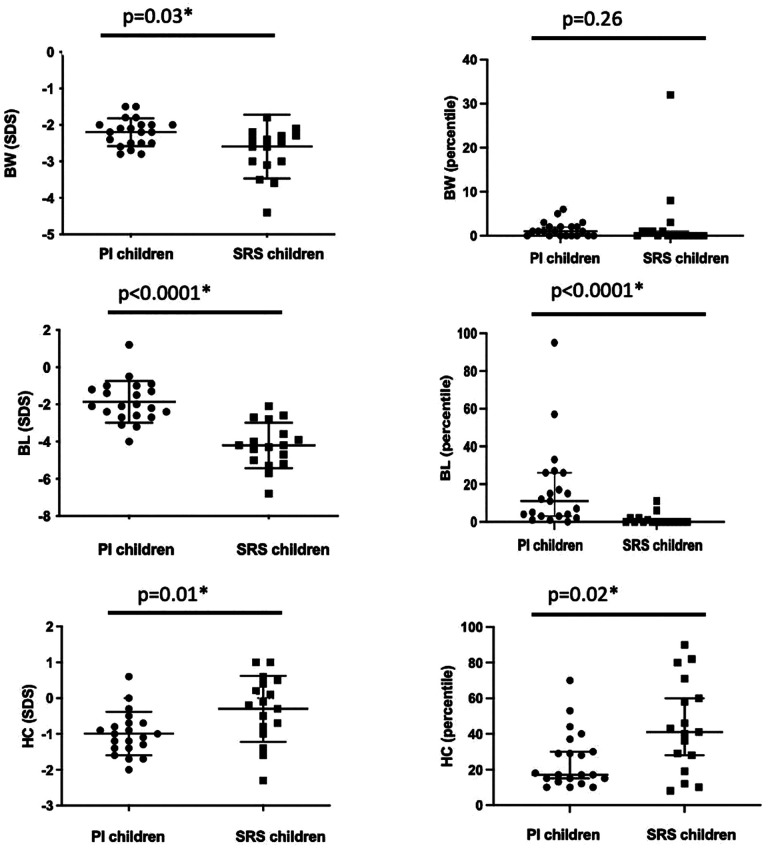
Comparison of the birth weight (BW), birth length (BL), and head circumference (HC) at birth in SDS and percentiles between the two groups. Bars represent the median and 95% confidence interval. *p < 0.05.

The biometric parameters at birth of children included in our study are presented in Figure 2 and supplemental data (Supplementary Table SD5). SRS children were significantly smaller at birth SDS (≤3.0 for BL in 77% of cases in the SRS group vs*.* 15% in the PI group, *p* = 0.0001). Ten patients of the SRS group (58%) had a SDS ≤4.0 for BL vs. none in the PI group (*p* < 10^−5^).

All SRS patients met the definition of relative macrocephaly as defined in the NH-CSS, in contrast to patients in the PI group (13/22 or 59% of cases, *p* = 0.01).

### Other clinical characteristics

Half of the SRS children (53%) required enteral feeding through a nasogastric tube. SRS children were more difficult to wean from their tube than children in the PI group, with a longer median duration of enteral feeding (18 vs. 10 days, *p* = 0.05) and a later recovery of birth weight (Day 7 vs*.* Day 4, *p* = 0.01).

Retrospective analysis of birth photographs revealed a protruding forehead in 73% of SRS children. Parents of SRS children described body asymmetry in 27% of them from birth.

Of the 17 SRS children, three had external genitalia abnormalities at birth: one had bilateral cryptorchidism and one had hypospadias associated with bilateral cryptorchidism.

One child had bilateral 5th finger clinodactyly, and another had syndactyly of the toes.

## Discussion

Here, we report, for the first time, that SRS children have early and severely altered foetal biometric parameters relative to SGA children due to PI. SRS children were smaller at birth (SDS ≤-3 for BL in 77% of cases in the SRS group vs*.* 15% in the PI group, *p* = 0.01), with relative macrocephaly, as defined by the NH-CSS, for 100% of the SRS children vs*.* 59% in the PI group (*p* = 0.01). The presence of uterine artery Doppler anomalies, a parameter classically used to orient the cause of FGR towards placental vascular insufficiency (1), did not rule out a diagnosis of SRS, as 23% of patients with SRS showed vascular anomalies.

The main limitation of our study was the small number of patients included. However, SRS is a rare disease and we still succeeded in collecting the complete data for 17 SRS children. To homogenise the SRS group, we included only patients with *H19*/*IGF2*: IG-DMR LOM, the most common molecular abnormality of SRS patients (8).

Children with SRS and those with SGA due to PI all had a relatively preserved HC at birth, but relative macrocephaly, as defined by the NH-CSS, was universal in the SRS group, in contrast to the PI group. The relative macrocephaly observed for children with SRS is hypothesised to be due to the biallelic expression of *IGF2* in the parts of the brain in which *IGF2* is not imprinted (16). Therefore, *IGF2*/*H19*: IG-DMR LOM on the paternal allele would have little impact on brain development and would explain why the median birth HC of children with SRS was close to the mean, with an SDS of −0.2. The mechanism of preservation of HC in the SGA fetus due to PI is different. Chronic intrauterine hypoxia and FGR induce an adaptive response of the fetus in which the cardiac output is redistributed to favour the vital organs, including the brain. Despite such an adaptive response, it is now apparent that brain sparing does not ensure normal brain development in growth restricted foetuses, who show a reduction in brain volume and total cell number and altered cortical grey matter and myelination (17).

Our study did not show a higher rate of medically assisted reproduction pregnancies for SRS children relative to PI children, probably because of the large amount of missing data for the SRS children (53%). However, recent studies suggest that medically assisted reproduction may alter genomic imprinting by interfering with gametogenesis and early preimplantation of the embryo (18).

Most SRS children underwent an invasive investigation to determine the cause of SGA. Indeed, IUGR due to PI usually has a late-onset and guidelines recommend investigating severe and early-onset IUGR (19). In our study, no cause had been found to explain IUGR among the SRS children. Currently, DNA methylation analysis of amniotic fluid spanning imprinted loci on chromosomes 7, 11, and 14 (the main molecular causes of SRS) is not validated, mainly because *H19/IGF2:-*DMR LOM varies between tissues or may have a mosaic distribution and thus might escape molecular detection (20). In cases of low-level mosaicism, a false-negative result may occur. On the other hand, a false-positive result is not excluded, because, thus far, there is no consensus on the target methylated DMRs in amniotic fluid in imprinting disorder testing (20). In our study, none of the SRS patients had had prenatal DNA methylation analysis.

Our study also showed that the morphological features of SRS, such as protruding forehead and body asymmetry, can be present from the neonatal period. When parents of SRS children were re-interviewed and asked for photographs of their children at birth, 73% were retrospectively identified with a protruding forehead and 27% had body asymmetry.

Finally, it is important to systematically retrieve the placental pathology report to confirm or refute the diagnosis of PI. In our study, no vascular lesions were described in the placenta of SRS patients. According to our results, a Japanese study of 138 patients with SRS reported a placental hypoplasia with mean placental weight −2.1 SDS (10). However, authors didn't realize histologic description of these placentas. In another Japanese study, Yamazawa et al. described a mean placental weight of −1.8 SDS in SRS children with 11p15 epimutation small placenta with hypoplastic chorionic villi frequently identified in histology (21).

## Conclusion

The diagnosis of SRS must be evoked in the neonatal period for newborns born SGA presenting a growth delay present from the second trimester of pregnancy with a SDS ≤-3 for BL and relative macrocephaly. Our study suggests the importance of expressing birth biometric parameters as a SDS, and not only percentiles, to allow neonatologists to diagnose relative macrocephaly (defined as a SDS ≥1.5 for HC at birth). Doppler anomalies should not systematically lead to a diagnosis of placental vascular insufficiency and a diagnosis of PI should be questioned in the absence of histologically confirmed placental vascular lesion. Other clinical signs in favour of a diagnosis of SRS, such as a protruding forehead and body asymmetry, may be present from birth, and have to been researched in neonate with severe SGA. Neonates with severe SGA, relative macrocephaly and absence of histologically confirmed placental vascular lesion have to been referred to expert center to discuss molecular investigation to allow an early diagnosis of SRS.

## Data Availability

The original contributions presented in the study are included in the article/Supplementary Material, further inquiries can be directed to the corresponding author.

## References

[1] Gordijn SJ Beune IM Thilaganathan B Papageorghiou A Baschat AA Baker PN Consensus definition of fetal growth restriction: a Delphi procedure. Ultrasound Obstet Gynecol. (2016) 48(3):333–9. 10.1002/uog.15884 26909664

[2] American College of Obstetricians and Gynecologists’ Committee on Practice Bulletins—Obstetrics and the Society forMaternal-FetalMedicin. ACOG practice bulletin no. 204: fetal growth restriction. Obstet Gynecol. (2019) 133(2):e97–109. 10.1097/AOG.0000000000003070 30681542

[3] Giabicani E Pham A Brioude F Mitanchez D Netchine I. Diagnosis and management of postnatal fetal growth restriction. Best Pract Res Clin Endocrinol Metab. (2018) 32(4):523–34. 10.1016/j.beem.2018.03.013 30086872

[4] Clayton PE Cianfarani S Czernichow P Johannsson G Rapaport R Rogol A. Management of the child born small for gestational age through to adulthood: a consensus statement of the international societies of pediatric endocrinology and the growth hormone research society. J Clin Endocrinol Metab. (2007) 92(3):804–10. 10.1210/jc.2006-2017 17200164

[5] Vanhaesebrouck A Vilain A Rey S Fresson J. Les maternités en 2016: résultats de l’enquête nationale périnatale (ENP). Revue D’Épidémiologie et de Santé Publique. (2018) 66:S54. 10.1016/j.respe.2018.01.126

[6] Nardozza LMM Caetano ACR Zamarian ACP Mazzola JB Silva CP Marçal VMG Fetal growth restriction: current knowledge. Arch Gynecol Obstet. (2017) 295(5):1061–77. 10.1007/s00404-017-4341-9 28285426

[7] DeChiara TM Efstratiadis A Robertsen EJ. A growth-deficiency phenotype in heterozygous mice carrying an insulin-like growth factor II gene disrupted by targeting. Nature. (1990) 345(6270):78–80. 10.1038/345078a0 2330056

[8] Wakeling EL Brioude F Lokulo-Sodipe O O’Connell SM Salem J Bliek J Diagnosis and management of Silver–Russell syndrome: first international consensus statement. Nat Rev Endocrinol. (2017) 13(2):105–24. 10.1038/nrendo.2016.138 27585961

[9] Azzi S Salem J Thibaud N Chantot-Bastaraud S Lieber E Netchine I A prospective study validating a clinical scoring system and demonstrating phenotypical-genotypical correlations in Silver-Russell syndrome. J Med Genet. (2015) 52(7):446–53. 10.1136/jmedgenet-2014-102979 25951829 PMC4501172

[10] Fuke T Mizuno S Nagai T Hasegawa T Horikawa R Miyoshi Y Molecular and clinical studies in 138 Japanese patients with Silver-Russell syndrome. PLoS One. (2013) 8(3):e60105. 10.1371/journal.pone.0060105 23533668 PMC3606247

[11] Hadlock FP Harrist RB Sharman RS Deter RL Park SK. Estimation of fetal weight with the use of head, body, and femur measurements–a prospective study. Am J Obstet Gynecol. (1985) 151(3):333–7. 10.1016/0002-9378(85)90298-4 3881966

[12] Mamelle N Grandjean H. Croissance fœtale à partir de l’étude AUDIPOG. I. Établissement de courbes de référence. J Gynecol Obstet Biol Reprod. (1996) 25:61–70. 8901304

[13] Usher R McLean F. Intrauterine growth of live-born Caucasian infants at sea level: standards obtained from measurements in 7 dimensions of infants born between 25 and 44 weeks. J Pediatr. (1969) 74(6):901–10. 10.1016/S0022-3476(69)80224-6 5781799

[14] Sempé A Pedron G Roy-Pernot M-P. Auxologie, Méthode et Séquences. Paris: Laboratoires 107 Théraplix (1979).

[15] Rolland-Cachera MF Cole TJ Sempé M Tichet J Rossignol C Charraud A. Body mass index variations: centiles from birth to 87 years. Eur J Clin Nutr. (1991) 45(1):13–21. 1855495

[16] Pham NV Nguyen MT Hu JF Vu TH Hoffman AR. Dissociation of IGF2 and H19 imprinting in human brain. Brain Res. (1998) 810(1–2):1–8. 10.1016/S0006-8993(98)00783-5 9813220

[17] Miller SL Huppi PS Mallard C. The consequences of fetal growth restriction on brain structure and neurodevelopmental outcome. J Physiol. (2016) 594(4):807–23. 10.1113/JP271402 26607046 PMC4753264

[18] Le Bouc Y Rossignol S Azzi S Brioude F Cabrol S Gicquel C Epigenetics, genomic imprinting and developmental disorders. Bull Acad Natl Med. (2010) 194(2):287–97.; discussion 297–300. 21166119

[19] Bamberg C Kalache KD. Prenatal diagnosis of fetal growth restriction. Semin Fetal Neonatal Med. (2004) 9(5):387–94. 10.1016/j.siny.2004.03.007 15691774

[20] Eggermann T Brioude F Russo S Lombardi MP Bliek J Maher ER Prenatal molecular testing for Beckwith-Wiedemann and Silver-Russell syndromes: a challenge for molecular analysis and genetic counseling. Eur J Hum Genet. (2016) 24(6):784–93. 10.1038/ejhg.2015.224 26508573 PMC4867462

[21] Yamazawa K Kagami M Nagai T Kondoh T Onigata K Maeyama K Molecular and clinical findings and their correlations in Silver-Russell syndrome: implications for a positive role of IGF2 in growth determination and differential imprinting regulation of the IGF2-H19 domain in bodies and placentas. J Mol Med (Berl). (2008) 86(10):1171–81. 10.1007/s00109-008-0377-4 18607558

