# Supplementary material for: Perinatal features of children with Silver-Russell syndrome due to 11p15 loss of methylation
Source: Front Pediatr. 2024 Apr 4;12:1367433. doi: 10.3389/fped.2024.1367433 (PMC11024461; doi:10.3389/fped.2024.1367433)
Supplement: Supplementary file 1 [file Datasheet1.docx]

**Supplemental data**

| **Clinical criteria** | **Definition** |
| --- | --- |
| SGA (birth weight and/or birth length | SDS ≤ -2.0 for gestational age |
| Postnatal growth failure | SDS ≤ -2.0 for height at 24 ± 1 months  Or  SDS ≤ -2.0 for height from midparental target height |
| Relative macrocephaly at birth | SDS ≥ 1.5 for head circumference at birth above the SDS for birth weight and/or length |
| Protruding forehead | Forehead projecting beyond the facial plane on a side view as a toddler |
| Body asymmetry | Leg length discrepancy (LLD) of ≥ 0.5 cm or arm asymmetry or LLD < 0.5 cm with at least two other asymmetrical body parts (one non-face) |
| Feeding difficulties and/or low BMI | SDS ≤ -2.0 for BMI at 24 months or use of a feeding tube or cyproheptadine for appetite stimulation. |

**Table SD1.** The Netchine-Harbison clinical scoring system (NH-CSS)

SGA: small for gestational age, SDS: standard deviation score, BMI: body mass index

|  | **SRS children**  **(N = 17)** |
| --- | --- |
| SGA (SDS ≤ −2 for BW and/or BL for gestational age) | 17/17 (100%) |
| Relative macrocephaly at birth | 17/17 (100%) |
| Postnatal growth failure | 17/17 (100%) |
| Feeding difficulties and/or low BMI | 15/16 (94%) |
| Protruding forehead | 11/15 (73.3%) |
| Body asymmetry | 13/16 (81.2%) |
| NH-CSS = 4 | 3/17 (18%) |
| NH-CSS = 5 | 7/17 (41%) |
| NH-CSS = 6 | 7/17 (41%) |

**Table SD2.** Clinical features and NH-CSS for SRS patients at the time of inclusion.

SGA: small for gestational age, BW: birth weight, BL: birth length, SDS: standard deviation score, BMI: body mass index, NH-CSS: Netchine-Harbison clinical scoring system.

|  | **PI Children (N = 21)** | **SRS Children**  **(N = 17)** | **p** |
| --- | --- | --- | --- |
| Term (WA) | 22.5  [21.4; 25.1] | 22.0  [21.4; 23.0] | 0.08 |
| EFW (grams) | 494  [333; 605] | 365  [292; 463] | < 0.0001*** |
| EFW (percentiles) | 13  [0; 95] | 0  [0; 13] | < 0.0001*** |
| BPD (millimetres) | \| 56  [46; 62] \| \| --- \| \|  \| | \| 52  [47; 58.40] \| \| --- \| | 0.04*** |
| BPD (percentiles) | 38  [0; 93] | 30  [1; 67] | 0.21 |
| HC (millimetres) | \| 201  [124; 222] \| \| --- \| \|  \| | \| 196  [180; 210] \| \| --- \| | 0.20 |
| HC (percentiles) | \| 40  [0; 87] \| \| --- \| \|  \| | 46  [15; 75] | 0.75 |
| AC (millimetres) | \| 175  [149; 191] \| \| --- \| \|  \| | 149  [134; 162] | < 0.0001*** |
| AC (percentiles) | \|  \| \| \| --- \| --- \| \|  \| \| \| 25  [1; 89] \| \|  \| | \| 2  [0; 22] \| \| --- \| | < 0.0001*** |
| FL (millimetres) | \| 38  [32; 43] \| \| --- \| \|  \| | \| 34  [30; 42] \| \| --- \| | 0.01*** |
| FL (percentiles) | 20  [0; 81] | 2  [0; 73] | 0.01*** |

**Table SD3.** Comparison of foetal biometric parameters at obstetric ultrasound performed at 22 WA between the two groups *p < 0.05

WA: week of amenorrhea, EPW: estimated foetal weight, BPD: biparietal diameter, HC: head circumference, AC: abdominal circumference, FL: femoral length, [xx; xx]: Min - Max

|  | **PI Children (N = 21)** | **SRS Children**  **(N = 17)** | **p** |
| --- | --- | --- | --- |
| Term (WA) | \| 33  [26; 36] \| \| --- \| \|  \| | \| 31  [24; 36] \| \| --- \| | 0.02*** |
| EFW (percentiles) | \| 8  [0; 65] \| \| --- \| \|  \| | \| 0  [0; 14] \| \| --- \| | < 0.0001*** |
| BPD (percentiles) | 21  [0; 75]   \|  \| \| --- \| \|  \| | \| 26  [1; 87] \| \| --- \| | 0.37 |
| HC (percentiles) | \| 41  [4; 88] \| \| --- \| \|  \| | \| 58.5  [21; 83] \| \| --- \| | 0.07 |
| AC (percentiles) | \|  \| \| \| --- \| --- \| \|  \| \| \|  \| \| \| 29  [2; 52] \| \| --- \| \|  \| \| | \| 2  [0; 21] \| \| --- \| | < 0.0001*** |
| FL (percentiles) | \| 9  [0; 82] \| \| --- \| \|  \| | \| 0.5  [0; 16] \| \| --- \| | 0.01*** |

**Table SD4.** Comparison of foetal biometric parameters at obstetric ultrasound performed between 31 and 33 WA between the two groups. *p < 0.05

WA: week of amenorrhea, EPW: estimated foetal weight, BPD: biparietal diameter, HC: head circumference, AC: abdominal circumference, FL: femoral length, [xx ; xx]: Min - Max

|  | **PI Children (N = 21)** | **SRS Children**  **(N = 17)** | **p** |
| --- | --- | --- | --- |
| BW (SDS) | \| -2.2  [-2.8; -1 .5] \| \| --- \| \|  \| | \| -2.5 \| \| --- \|   [-4.4; - 0.0] | 0.03*** |
| BW (percentile) | \| 1  [0; 6] \| \| --- \| \|  \| | \| 0 \| \| --- \|   [0; 32] | 0.26 |
| BL (SDS) | \| -2.1  [-4.0; - 1.2] \| \| --- \| \|  \| | \| -4.2 \| \| --- \|   [-6.8; - 2.1] | <0.0001*** |
| BL (percentile) | \| 11  [0; 95] \| \| --- \| \|  \| | \| 0 \| \| --- \|   [0; 11] | <0.0001*** |
| HC at birth (SDS) | \| -1.0  [-2.0; 0.60] \| \| --- \| \|  \| | \| -0.2 \| \| --- \|   [-2.3; 1.0] | 0.01*** |
| HC at birth (percentile) | \| 17  [10; 70] \| \| --- \| \|  \| | \| 41 \| \| --- \|   [8; 90] | 0.02*** |
| SDS difference between HC at birth and BL | 0.95  [-2.4; 2.6] | 3.60  [2.6; 6.6] | <0.0001*** |
| SDS difference between HC at birth and BW | 1.15  [0.1; 3.3] | 2  [0.4; 3.4] | 0.01*** |
| SDS difference between BW and BL | -0.05  [-1.5; 3.2] | 1.7  [-0.3; 3.6] | 0.01*** |

**Table SD5.** Comparison of birth biometrics between the two groups * p < 0.05

BL: birth length, BW: birth weight, HC: head circumference, SDS: standard deviation score, [xx; xx]: Min - Max
